# Double Fault Detection of Cone-Shaped Redundant IMUs Using Wavelet Transformation and EPSA

**DOI:** 10.3390/s140203428

**Published:** 2014-02-19

**Authors:** Wonhee Lee, Chan Gook Park

**Affiliations:** 1 Department of Mechanical and Aerospace Engineering, ASRI, Seoul National University, Seoul 151-741, Korea; E-Mail: clever212@snu.ac.kr; 2 Department of Mechanical and Aerospace Engineering, Institute of Advanced Aerospace Technology, Seoul National University, Seoul 151-741, Korea

**Keywords:** extended parity space approach, fault detection and isolation, inertial sensor, redundant IMU, wavelet transform

## Abstract

A model-free hybrid fault diagnosis technique is proposed to improve the performance of single and double fault detection and isolation. This is a model-free hybrid method which combines the extended parity space approach (EPSA) with a multi-resolution signal decomposition by using a discrete wavelet transform (DWT). Conventional EPSA can detect and isolate single and double faults. The performance of fault detection and isolation is influenced by the relative size of noise and fault. In this paper; the DWT helps to cancel the high frequency sensor noise. The proposed technique can improve low fault detection and isolation probability by utilizing the EPSA with DWT. To verify the effectiveness of the proposed fault detection method Monte Carlo numerical simulations are performed for a redundant inertial measurement unit (RIMU).

## 1. Introduction

The inertial measurement unit (IMU) is an essential part of the attitude heading reference system (AHRS) and inertial navigation system (INS). Generally, it consists of three gyroscopes and accelerometers, respectively, that are arranged orthogonally. Each sensor operates independently, hence it is impossible to detect the sensor fault of other axes. A fault in the navigation sensors can be a fatal blow to the total system. Therefore, systems that must have a high reliability like satellites, missiles and aircraft use the hardware redundant type fault detection isolation (FDI) method. The redundant IMU dealt with this paper is an example of a hardware redundant method. The redundant IMU has several redundant sensors and the configuration affects the performance of the FDI [1–5]. In this paper, the redundant inertial measurement unit (RIMU) used seven sensors, which are arranged in a cone shape. Generally, it this known to be a good option for fault detection.

The FDI process is used to verify the availability of sensor signals. Several FDI methods were introduced in past research [6–12]. For example, the parity space approach (PSA), generalized likelihood ratio test (GLT) and optimal parity vector test (OPT) are suggested [13–25]. They are generally used for single fault detection. These FDI methods are difficult to extend for double fault detection and isolation. The effect of double faults is a mixed form of single faults. Therefore, FDI performance degradation occurs when single fault detection methods are applied for double faults. The proposed FDI technique is based on an extended PSA concept, which was proposed by Potter and Suman [22–26]. EPSA has several PSA layers, because the PSA can be only used one time in FDI [24–26]. If we want to know which sensor is faulty when a double fault has occurred, we must use multiple layers. Therefore, conventional EPSA has issues under some conditions. The EPSA is not suitable when the fault direction angle between the faulty sensors is large. Additionally, the sensor noise affects the FDI performance of EPSA. Hence, we introduce a modified EPSA by using together a discrete wavelet transform. The discrete wavelet transform can decompose the signal according to scale. The multi-resolution signal decomposition is useful to remove a specific frequency signal [27–30]. In this paper, decomposed sensor data is used for inline monitoring and high frequency noise cancellation. Adoption of a DWT in EPSA introduced a great improvement for FDI success probability by reducing the effect of sensor noise in the parity vector.

This paper is organized as follows: Section 2 deals with the classification of faults. Section 3 describes how to detect and isolate double faults using EPSA. In Section 4, we will explain the signal decomposition technique using DWT. Section 5 describes the fault detection and isolation algorithm using the modified EPSA, and a proposed FDI algorithm simulation results are shown in Section 6. Finally, Section 7 presents the conclusions.

## 2. Fault Classification

Generally, the sensors that are used in inertial navigation devices rarely suffer faults. Thus, two kinds of fault types were considered: single and double faults. A single fault means there is only 1 faulty sensor. The FDI process to determine the faulty sensor is identical. On the other hand, the FDI process of double faults was selected in accordance with its type, because double faults cause a difference of the parity vector. In PSA-based FDI methods, the size of the parity vector is the FDI criterion. If they are not classified before FDI, it is difficult to detect and isolate double faults correctly. Firstly, the types of sensor faults that might occur in RIMU are considered, and then the detection and isolation of the fault in accordance with the type are described.

The inertial sensor fault can be modeled according to Table 1. We assume that the maximum number of faulty sensors that can occur at the same time is 2. The double faults are classified into 2 types according to the fault direction angle.

**Figure 1.**
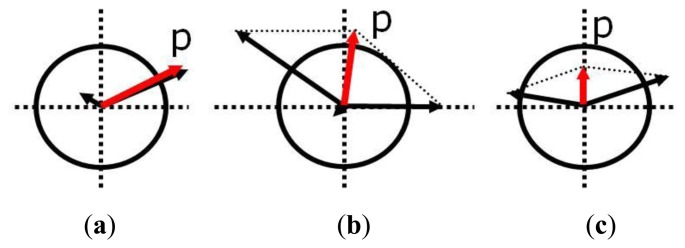
Types of sensor fault. (**a**) fault A. (**b**) fault B. (**c**) fault C.

Figure 1 is the fault type introduced in Table 1. The circle refers to a threshold that is the criterion for fault detection. The magnitude and direction of the arrows refer to the fault size and fault direction, respectively [26]. By using this fault classification, the FDI technique design and simulation were performed.

## 3. Extended Parity Space Approach

### 3.1. Parity Space Approach

PSA is one of the most commonly used methods for fault detection and isolation. A parity vector that is independent and sensitive to the fault is defined as a state variable. The parity vector derived from the matrix *V* and matrix *V* was calculated by the measurement matrix *H*. These were defined by Potter and Suman [14]. Matrix *V* is a trapezoidal matrix that satisfies the following conditions:

(1) VH=0

(2) $$VV^{T}=I_{l-n}$$

(3) $$V={\left[\begin{array}{cccc} v_{1} & v_{2} & \cdots & v_{l-n} \end{array}\right]}^{T}=\left[\begin{array}{cccc} v_{c1} & v_{c2} & \cdots & v_{cl} \end{array}\right]$$

where *l* and *n* are the dimension of measurement output vector and the number of RIMU sensors, respectively, 1 ≤ *n* < *l*. *I_l_*_−_ *_n_* is an identity matrix with (*l* − *n*) × (*l* − *n*) dimensions, and the dimension of *V* is (*l* − *n*) × *l*. $v_{i}^{T}$ is the *i^th^* row vector of *V* and *ν_ck_* is the *k^th^* column vector of *V*.

**Definition 3.1**: The column space of matrix *V* is defined as the “parity space” of the measurement matrix *H*.

**Definition 3.2**: The parity vector is defined by:

(4) $$\begin{array}{ll} p & =Vm\\ & =V(Hx+ɛ+f)\\ & =Vɛ+Vf \end{array}$$

(5) m=Hx+ɛ+f

(6) $$p={\left[\begin{array}{cccc} p_{1} & p_{2} & \cdots & p_{l-n} \end{array}\right]}^{T}$$

where *p* is the *l* − *n* dimensional vector. The parity vector is the projection of the measurement *m* onto the parity space and is independent of the state variables but dependent on the sensor fault and noise. Therefore, the F/N (fault to noise) ratio is important to analyze the FDI performance. *m* is the *l* × 1 measurement output vector of sensor, *H* is the *l* × 1 observation matrix of rank *n* to be determined by sensor configuration, the state *x* is the *l* × 1 true value of the measured variable and *ε* is the *l* × 1 Gaussian measurement noise vector, *f* is the fault signal vector and the type of fault is modeled as constant bias. Although this assumption may have some problems, this is a valid and common assumption in that the bias type fault has a great effect on the system. In Equation (6), *p_i_* is called a parity equation.

**Definition 3.3**: The column of *V*, *ν_ck_*, is projections of the *k* -th measurement directions on the *c*-th parity space and is called the *c*-th sensor fault direction since the fault on the *k* -th measurement *m_k_* implies the growth of the parity vector *p* A vector *ν_ck_m_k_* on the fault direction of *ν_ck_* is called a fault direction vector [16]. The fault direction angle is defined the angle between 2 different fault direction vectors.

#### (1) Fault detection by using PSA

*p^T^p* has *χ*^2^ distribution with *l* − *n* DOF (degrees of freedom) and is used as the fault detection function, *i.e.*, *FD* = *χ*^2^ = *p^T^p*. If the probability of false alarm is *α*, *T* to satisfy *P*(*χ*^2^ > *T*) = *α* is determined as the threshold value from the *χ*^2^ distribution table, where *P* is the probability function. The fault is detected by checking the value of *p^T^p*. If, it means the fault does not occur. If *p^T^p* > *T*, the sensor module has the fault. *p^T^p* ≤ *T*

#### (2) Fault isolation by using PSA

The fault isolation function is defined as $FI_{k}=v_{ck}^{T}p/\left\|v_{ck}\right\|$. This function shows the value to be obtained as projecting the parity vector along the fault direction of each sensor. The number of *FI* functions is *l*. The sensor that related a maximum *FI* value is considered as the faulty sensor. For example, if *FI_k_* is the maximum, the *k^th^* sensor is isolated as the faulty sensor. Detailed information about PSA can be easily obtained [16].

### 3.2. EPSA Configuration Based on PSA

**Figure 2.**
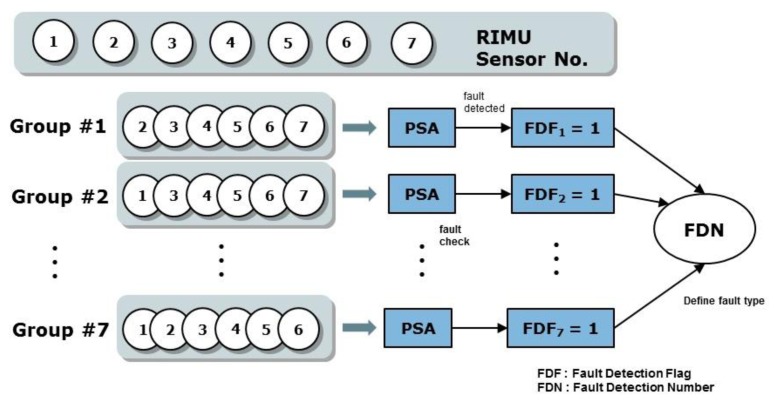
Fault detection using EPSA.

**Figure 3.**
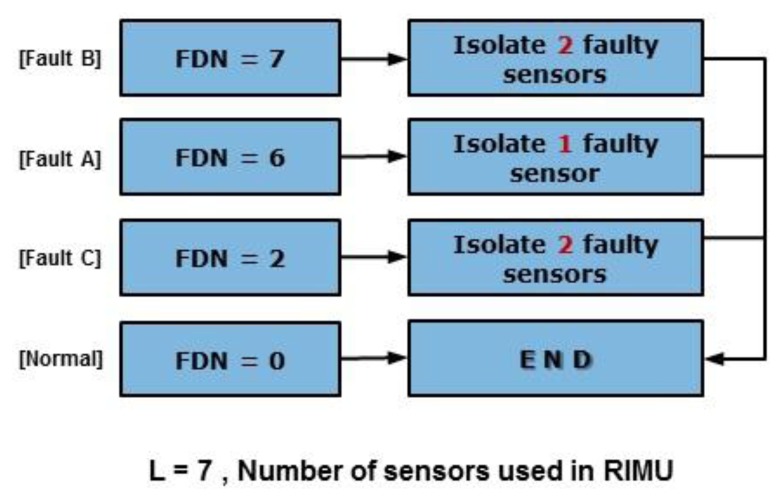
Fault isolation according to the type.

The fault detection concept of EPSA is basically the same as that of the PSA. However, with PSA it is only possible to find single faults. EPSA is designed to solve the single and double faults detection problem. To detect the double faults, seven sensor groups that are composed of six sensors each are used. Each sensor group is used to determine whether the fault occurred or not. The fault detection number of the sensor group is used to decide which type of fault happened. Figure 2 shows the EPSA process for fault detection. To ascertain whether each sensor group has the fault, we check the size of the parity vector using Equation (4). The Fault Detection Number (FDN) is calculated by adding the number of faulty groups. As a result, this FDN value refers to the type of fault that occurred. If the FDN is 6, it means a single fault occurred. If FDN is 2 and 7, it means type C and B double faults occurred, respectively. After the fault detection, a fault isolation process is performed according to the fault type as indicated in Figure 3.

#### (1) FDI of fault type A

This is the single fault case. Because one sensor among the seven sensors in the RIMU has the fault, the fault is detected in six sensor groups. FDN is 6, and the faulty sensor is judged using the sensor number that is not comprised in the non-faulty sensor group.

#### (2) FDI of fault type B

This is one of the double fault cases. If a fault type B occurred, it means that the sum of each parity vector is bigger than the threshold. One of double faults also has a parity vector that as big as the threshold. Hence, the magnitude of the parity vector is bigger than the threshold, no matter if the double faults are in the same or different sensor group. FDN will be 7 when a type B fault occurred.

#### (3) FDI of fault type C

This is another type of double fault in RIMU. Type C faults happen rarely. This fault causes RIMU performance degradation problems because it is difficult to detect this fault when the conventional FDIR methods are utilized. EPSA can detect this fault by using the FDN for FDI. When the double fault occurs, the fault directions and magnitudes of the faulty sensors are important. If the fault direction angle between the faulty sensors is over 120 deg, it could be a candidate of fault type C. Because the sum of the fault direction of sensor groups that have two faulty sensors is smaller than the fault detection threshold, fault type C is detected by checking the magnitude of the parity vector at a sensor group containing a single faulty sensor. When the faulty sensors are in the same sensor group, the parity vector is smaller than the threshold. Otherwise, when the faulty sensors are in different sensor groups, the parity vector is bigger than the threshold because of the single-faulty sensor effect. Therefore, when FDN is 2, it means that a type C fault occurred.

## 4. Signal Decomposition

The magnitude of the parity vector is affected by sensor noise and faults as indicated in Equation (4). Therefore, an effective noise canceling technique helps to increase the FDI performance. In this paper, noise canceling was performed by using DWT that was already proved in many cases [29].

Generally, the Fourier transform has been used for signal processing in the frequency domain. However, it is difficult to know the time-frequency relationship. The Fourier transform is less useful for analyzing non-stationary signals whose frequency contents change with time because it is difficult to recognize the signal in a specific frequency. To overcome this difficulty, the windowed Fourier transform was introduced. Even with the windowed Fourier transform, there is a weakness with the multi-scaled signal analysis because the resolution of the window function is constant over the entire frequency bandwidth. The wavelet transform, which has been developed for image processing and signal analysis [27], can provide time-frequency localization. The performance of wavelet transforms is better than the windowed Fourier transforms in the sense that it can zoom in on the high frequency signal for a short duration or zoom out on slow oscillations. Thanks to these characteristics, the wavelet transform can be used for analyzing non-stationary abnormal signals at the time of the sensor's fault. The DWT is defined as follows [29]:

(7) $$W_{\psi }f(b,a)=\int_{-\infty }^{\infty }f(t)\psi_{b,a}(t)dt$$

where the mother wavelet is:

(8) $$\psi_{b,a}(t)=\frac{1}{\sqrt{a}}\psi \frac{t-b}{a}$$

By selecting *a* and *b* properly, the dilated mother wavelets constitute an orthonormal basis. In this paper, the Daubecheise2 wavelet is used that is generally used in the mechanical system for fault detection [28].

Orthonormal properties of DWT enable multi-resolution signal decomposition, which projects a signal step by step into the dyadic frequency band in a manner similar to a filter bank algorithm. Starting from an original sensor signal, the first step of multi-resolution signal decomposition is to produce an approximate coefficient and detail coefficient. These vectors are obtained by convolving the sensor signal with a low-pass filter for ‘the approximations’, and with high-pass filter for ‘the details’. The computation load becomes smaller at the higher scale because the number of sample in each sub-band cuts down to half of that in the previous sub-band. The detailed version of the signal contains noise as well as fault information such as sharp edges, transitions, and jumps at the bias, drift, spike faults, and so on.

**Figure 4.**
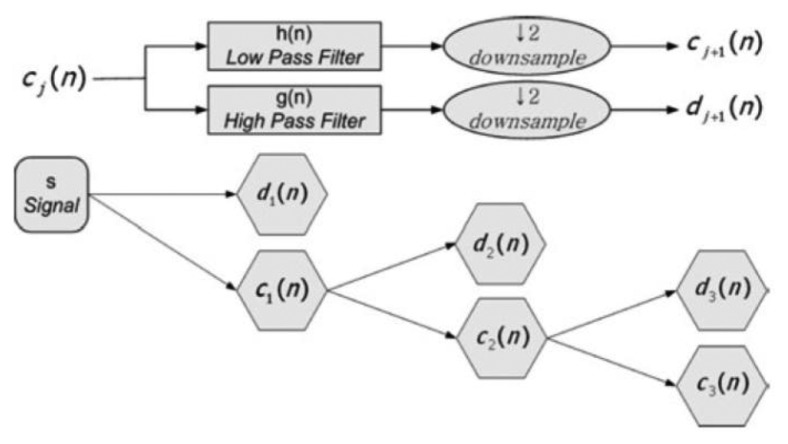
Signal decomposition using wavelet transform.

Let *c*_0_(*n*) be a discrete-time sensor signal at the discrete time step *n*. The sensor signal can be decomposed into a smoothed version *c_i_*(*n*) and detailed version *d*_i_(*n*) at scale as shown in Figure 4:

(9) $$c_{j+1}(n)=\overset{\infty }{\underset{k=-\infty }{\text{∑}}}k\left(k-2n\right)c_{j}\left(k\right)$$

(10) $$d_{j+1}(n)=\overset{\infty }{\underset{k=-\infty }{\text{∑}}}g\left(k-2n\right)c_{j}\left(k\right)$$

where *h*(*n*) and *g*(*n*) are the low-pass and high-pass filter coefficients, respectively, which can be determined by the following two-scale relation to the scaling function *φ*(*t*) and the wavelet function *ψ*(*t*) [29].

(11) $$\phi (t)=\sqrt{2}\overset{\infty }{\underset{n=-\infty }{\text{∑}}}h(n)\phi (2t-n)$$

(12) $$\psi (t)=\sqrt{2}\overset{\infty }{\underset{n=-\infty }{\text{∑}}}g(n)\phi (2t-n)$$

where *g*(*n*) = (−1)^1−^*^n^h*(1−*n*). If the scaling function *ϕ*(*t*) is defined as the signal basis, the low-pass filter coefficients *g*(*n*) and the wavelet function *ψ*(*t*) can be successively designed as in [25].

## 5. Modified Extended Parity Space Approach

**Figure 5.**
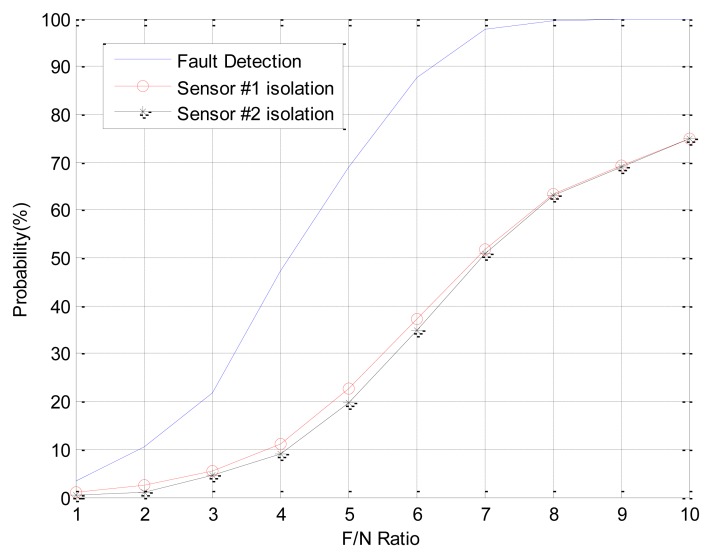
FDI probability of double faults: sensor 1 and sensor 2.

The FDI performance of EPSA is good for single faults and double faults. However when the fault direction is bigger than 120 deg such as in fault type 3, the FDI performance of EPSA is decreased. The fault isolation probability of EPSA is low when the fault direction angle is large. Table 2 shows the fault direction angle of double faults between sensor 1 and the other sensor. Fault C can be present when double faults occur in sensor 1 and 2 or sensor 1 and 7. The FDI performance could be low if double faults occur in sensor 1 and 2. Figure 5 shows the result of double fault detection and isolation, which have a large fault direction angle: sensor 1 and 2 are faulty sensors. While the fault detection probability is high, the fault isolation probability is low. This is caused by incorrect fault isolation because of a large fault direction angle. To prevent the FDI performance degradation, a modified-EPSA that consists of DWT and EPSA was proposed. The modified-EPSA enhances the fault detection and isolation probability, which is shown. As we use the DWT with EPSA, there is a possibility of improving the performance of the sensor fault FDI because we can separate the high frequency noise from the signal. It is possible to check the faulty sensor candidate one more time, and by using sensor data, change the quantity information to improve the FDI efficiency. As a result, FDI probability is increased by using the proposed FDI method. Especially, the fault isolation probability increased in faults type B and C.

### 5.1. DWT Combined EPSA

**Figure 6.**
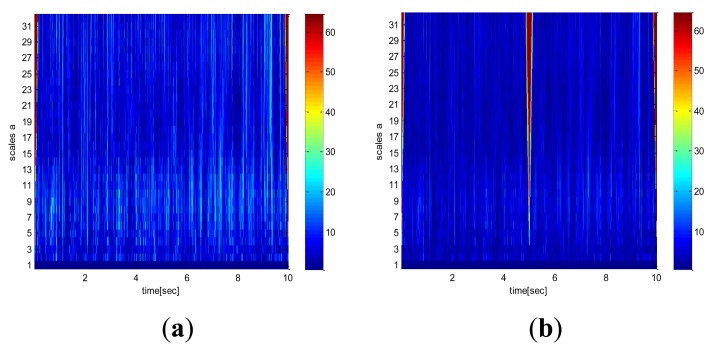
Sensor signal analysis result using the wavelet transform. (**a**) fault-free case. (**b**) fault case.

**Figure 7.**
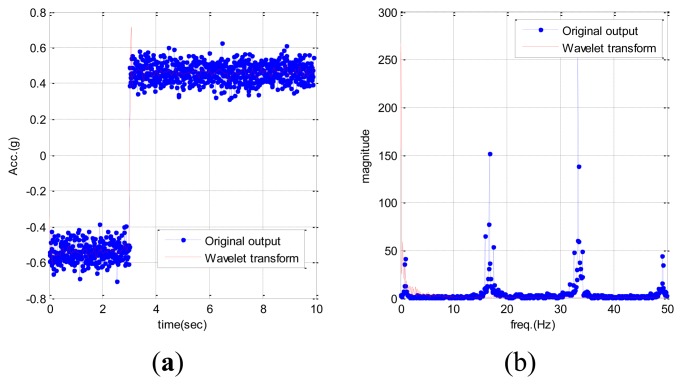
Reconstructed sensor signal. (**a**) time domain. (**b**) frequency domain.

The EPSA is mainly used for RIMU double fault detection and isolation. The DWT is applied in order to improve the EPSA results and minimize the effects of high frequency sensor noise. As we mentioned above, DWT has a peculiar property that separates the high frequency and low frequency signals. Therefore, it is useful to reduce the sensor noise effect for FDI performance. The parity vector used in EPSA is comprised of fault and noise terms as shown in Definition 3.2. The magnitude of this parity vector becomes the criterion to determine the location of the sensor fault. Hence, noise cancellation is very important for high performance. The conventional EPSA does not have a sensor noise cancellation method, therefore, the FDI performance can be decreased because of measurement noise. DWT can be a solution for FDI performance degradation caused by noise. Figure 6 is the signal analysis result using wavelet transform. Faults cause a specific effect, and DWT can detect the fault effect on the signal. With this technique it is possible to remove the noise by isolating the high pass noise components successfully. Consequently, more robust double fault detection and isolation method can be designed by using an additional noise cancelling step. Furthermore, the DWT can detect the sensor fault by itself. As indicated in Figure 5, big differences in the time-frequency analysis of sensor signals were found because a sudden change of sensor data caused the frequency changes. Moreover, more fault-dependent parity vector is acquired by using the noise cancelled sensor signal like in Figure 7. De-noised sensor signals allow for better double fault FDI performance.

### 5.2. FDI Using the Modified EPSA

The sensor fault is like a bias on the output signal because it causes a sudden change in the time domain. The sudden change appears at a relatively high frequency. Using the DWT, the sensor signal is decomposed in three steps. The signal that has the high frequency component is used for fault detection. The sensor fault is defined when the sensor signal has a value greater than the threshold. There are multiple methods for selecting a threshold. In this paper, the sensor signal variance is used. Additionally, the EPSA FDI algorithm is used as a main FDI method. It is not the same as EPSA, because the modified-EPSA uses a signal where the high frequency noise has been removed. This is the main difference between EPSA and modified-EPSA. High frequency noise is naturally removed by decomposing of sensor signal using DWT. Thanks to the reconstructed sensor signal, the FDI performance of RIMU becomes better than when the original sensor signal is used. The fault detection process is identical to the EPSA.

**Figure 8.**
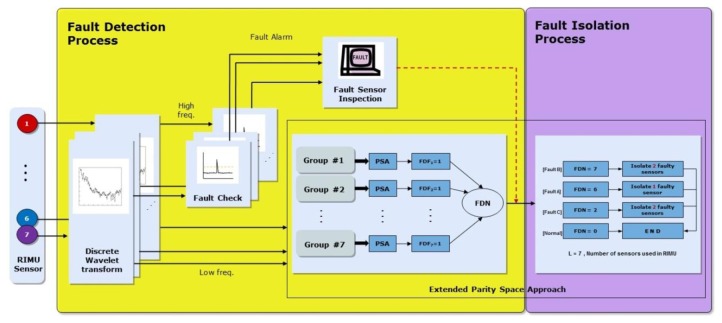
FDI scheme using modified EPSA.

**Figure 9.**
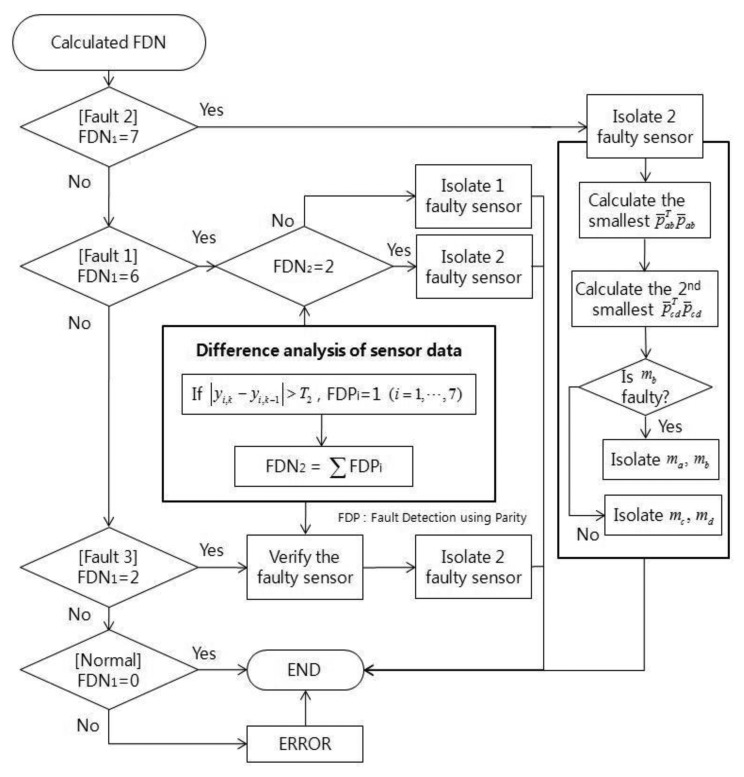
Fault isolation flow chart.

The fault isolation process is utilized in the EPSA isolation process and the different sensor data over time. FDN, which is calculated by the EPSA, has a different value according to fault type. Therefore, if an accurate FDN value can be calculated, the probability of the fault isolation process is increased. However, it is difficult to get that accurate number. For example, fault type C must have FDN 5. In many cases, it has a 6, which refers to the double fault, since a big fault direction angle over 120 deg is classified as a single fault. To overcome this problem, the difference between the sensor data over time is checked to recognize fault types one more time. The sensor data is effective at verifying fault detection results. Figures 8 and 9 are conceptual diagrams of the proposed FDI scheme, where each figure shows the details of the entire FDI process of the modified-EPSA and the fault isolation strategy for double faults. First of all, DWT is performed for every sensor signal, and the reconstructed sensor signals after DWT are used as a measurement to calculate the parity vector. FDF is computed using PSA for the every sensor group. The fault type was classified using the sum of FDF: FDN. The fault isolation process is focused on the classification of fault type by using the parity equation, *i.e.*, the differences in sensor signals over time.

## 6. Simulation

**Figure 10.**
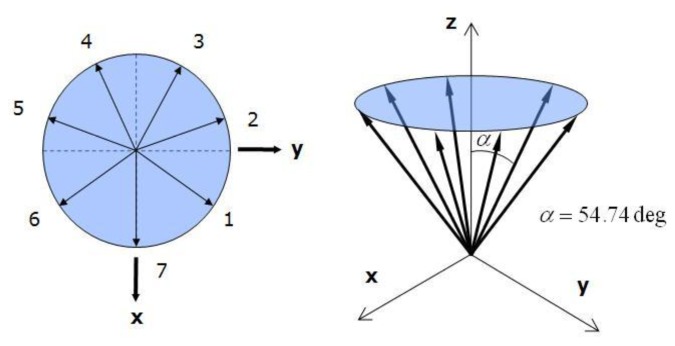
Redundant IMU configuration with seven sensors.

The RIMU has several redundant sensors, and its sensor configuration affects the FDI performance. In this paper, seven sensors were arranged in a cone shape, which is known to be good for fault detection. Figure 10 shows the specifications of the RIMU configuration. Each sensor tilts 54.74deg against the z-axis and is configured having an equal angle on the x-y plane.

The inertial sensor error model is represented by the misalignment error, bias and scale factor, which are the main sources of error for general IMU:

(13) m=MHx+b+ɛ+f

where *m* is the measurement of gyros and accelerometers in the redundant IMU (*m* ∈ *R^l^*^×1^). *M* is the misalignment error matrix (*M* ∈ *R^l^*^×1^), *H* is the measurement matrix that has consisted the direction vectors of each sensor (*H* ∈ *R^l^*^×3^), the state *x* is the input angular velocity or acceleration in the body coordinate (*x* ∈ *R*^3^), *b* is the sensor bias (*b* ∈ *R^l^*), *ε* is the measurement noise (*ε* ∈ *R^l^*), and *f* is the additive fault of the inertial sensor that has an effect only on the sensor bias (*f* ∈ *R^l^*). Although the assumptions about sensor faults may have some problems, this is a valid and common assumption since the bias type fault has a meaningful effect on the system.

Measurement matrix *H* is defined by the geometrical configuration, *H* can be like Equation (14) considering the seven cone-shaped sensors:

(14) $$H=\left[\begin{array}{rrr} 0.5091 & 0.6384 & 0.5774\\ -0.1817 & 0.7960 & 0.5774\\ -0.7356 & 0.3543 & 0.5774\\ -0.7356 & -0.3543 & 0.5774\\ -0.1817 & -0.7960 & 0.5774\\ 0.5091 & -0.6384 & 0.5774\\ 0.8165 & 0.0000 & 0.5774 \end{array}\right]$$

To verify the performance of the proposed FDI algorithm, the RIMU accelerometer outputs are used as shown in Figure 10. As to the use of the Monte Carlo method, the probabilistic approach for FDI is possible to find how the proposed FDI algorithm is better than the conventional algorithm. The simulations are repeated 1,000 times for each F/N ratio, and the fault is inserted in the sensor measurement according to the 1 to 10 F/N ratio. The FDI performance is presented based on the fault direction angle of double faults case: over 120deg or not. Table 3 is the FDI simulation result of double faults using conventional EPSA. If all of faults were detected, the fault detection number is 10,000 and the isolation number of double fault sensors is also 10,000, because the simulation is performed 1,000 times from F/N ratio 1 to 10. The FDI performance of EPSA seems consistent with the expectation that the fault direction angle will correlate significantly with FDI performance. The worst FDI cases were present when the double faults occurred at sensors 1, 2 and 1, 7, respectively, because the fault direction angle is larger than in the other cases. Fault C is also detected approximately 100 times. Otherwise, for the double faults cases which occurred at sensors 1, 4, we have a good performance too. Based on this performance analysis, modified EPSA simulation was performed.

**Figure 11.**
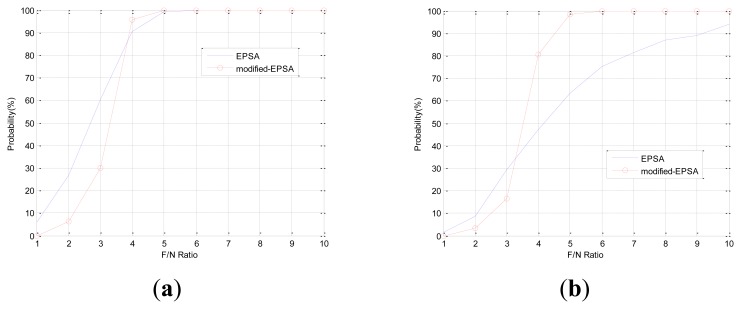
(CASE1) Double faults detection and isolation performance of sensor 1. (**a**) Detection probability. (**b**) Isolation probability.

**Figure 12.**
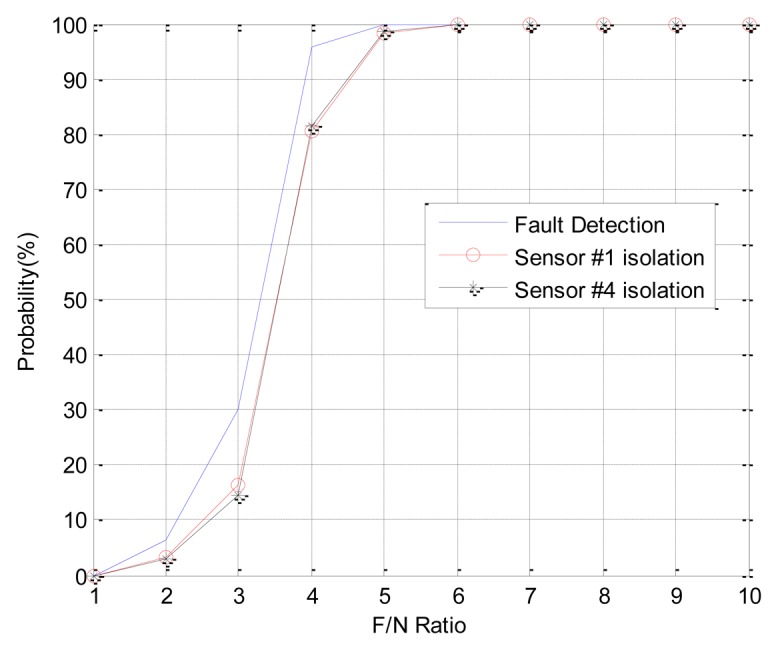
(CASE1) Double faults detection and isolation performance of sensor 1 and 4.

In Case 1, double faults occur at sensors 1 and 4, which have the best FDI performance. The angle of fault direction between the two faults is 78.43deg, and the effect of noise on the signal is not at the critical point. Figure 11 shows the successful FDI probability of sensor 1 in Case 1. The modified-EPSA can detect two faulty sensors 100% of the time and 98.4% of double faults are isolated successfully when the F/N ratio is bigger than 5. The fault isolation performance was good, and there is 20% improvement in fault isolation probability if we use EPSA with discrete wavelet transform. When the F/N ratio is smaller than 3, the conventional EPSA is better than the proposed FDI algorithm. However, it is meaningless because it can be caused by sensor noise. In other words, it is impossible to recognize whether it is a real fault or not. Figure 12 is the FDI result of sensor 1 and 4. Despite the double faults occur, the fault detection and isolation probability is high. Table 4 shows the fault isolation result according to F/N ratio in case 1. As the F/N ratio becomes large, incorrect fault sensor isolation probabilities were rapidly reduced.

**Figure 13.**
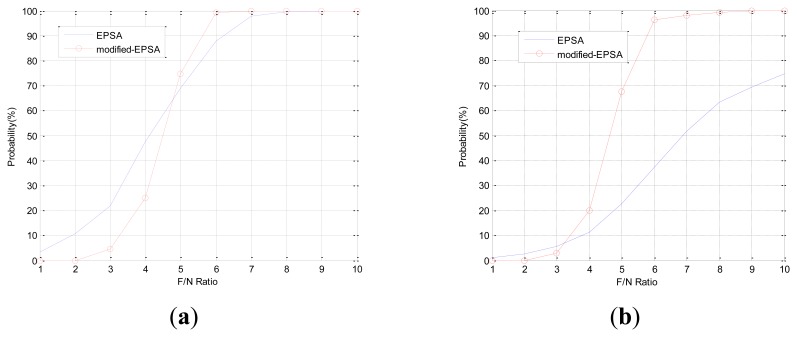
(CASE2) Double faults detection and isolation performance of sensor 1. (**a**) Detection probability. (**b**) Isolation probability.

**Figure 14.**
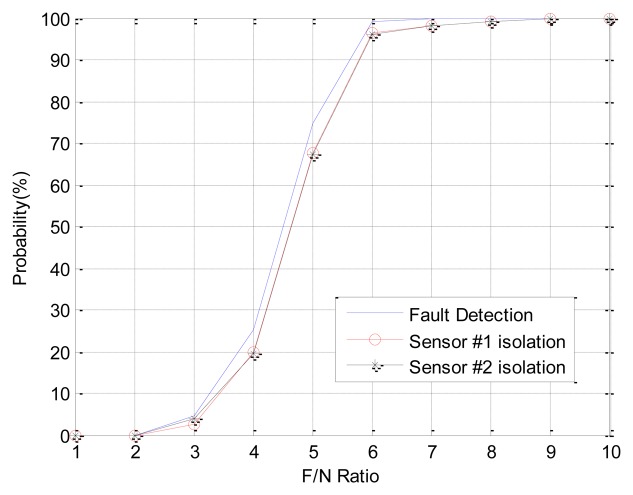
(CASE2) Double faults detection and isolation performance of sensor 1 and 2.

In Case 2, double faults occurred at sensors 1 and 2, which are neighbors. When double faults occur at two neighbor sensors, the magnitudes of faults are small. It is rarely possible to detect the fault using conventional EPSA. If the noise cancellation method is adopted, the modified-EPSA is able to overcome this problem. Figures 13, 14, and Table 5 show the FDI performance of sensor 1 in case 2. Fault detection and isolation probability is lower than the FDI probability of case 1 because of the small size of the parity vector. Hence, this is caused by the fault direction angle. When the F/N ratio is 5, the probability of fault detection and isolation are 74.6% and 67.5% with the modified-EPSA, respectively. The efficiency is not good, but it brings about an improvement, which is relatively remarkable when compared to the original EPSA. In particular, when the F/N ratio is 6, it is possible to detect and isolate 99.2% and 96.3%, respectively. Meanwhile the conventional FDI method can only detect 87.8% of faults and 37.8% of fault isolation. This difference is 11.4% and 58.5%. Cases 1 and 2 verified the effectiveness of the proposed FDI technique using a probabilistic approach.

## 7. Conclusions

This paper proposed the DWT combined with EPSA FDI method for RIMU. RIMU is configured into a cone shape using seven inertial sensors considering FDI performance. Double faults can occur in the RIMU, which are normally modeled as three different types: single and two different types of double faults. Double faults were classified using the angle of each fault direction. The sensor noise and fault direction angle of two faulty sensors affect the FDI performance when the EPSA is used. Therefore, the DWT was adopted for the EPSA method to mitigate the noise. When we use a discrete wavelet transform for noise cancelling, the modified-EPSA shows higher performance than the conventional method. When the double faults occur at sensors 1 and 4, the proposed method is better for the fault detection and isolation, which are 31% and 75.7%, respectively, when the F/N ratio is 5. Also, the modified-EPSA shows a maximum 10.4% and 59% FDI performance improvement when the F/N ratio is 6, when the double faults occurred at sensors 1 and 2.

## Figures and Tables

**Table 1.** Fault classification.

**Type**	**Fault Type Features**
Fault A	Single fault: the fault occurred in just one sensor
Fault B	Double faults: the fault occurred in two sensors
Fault C	Double faults: the fault occurred in two sensors. However, it is impossible to detect because of the RIMU geometry condition

**Table 2.** Fault direction angle between double faults.

**Numbers of Faulty Sensor**	**1-2**	**1-3**	**1-4**	**1-5**	**1-6**	**1-7**
Fault direction angle (deg)	124.2	97.97	78.43	78.43	97.97	124.2

**Table 3.** Conventional EPSA FDI performance.

Faulty Sensor Number		**1, 2**	**1, 3**	**1, 4**	**1, 5**	**1, 6**	**1, 7**

Fault direction angle (deg)		124.18	97.97	78.43	78.43	97.97	124.18
Fault detection number	Fault A	1,494	1,577	1,227	1,210	1,753	1,862
	Fault B	4,778	5,454	6,528	6,501	5,209	4,191
	Fault C	100	56	75	67	76	107

Fault isolation number	# 1	**4,058**	**4,824**	**6,459**	**6,652**	**4,341**	**3,688**
	# 2	**3,946**	1,962	538	195	686	1,200
	# 3	1,538	**4,769**	500	46	92	184
	# 4	194	428	**6,477**	196	137	263
	# 5	366	123	206	**6,543**	373	181
	# 6	245	75	47	346	**4,208**	1,299
	# 7	903	396	206	368	2,486	**3,643**

**Table 4.** Modidfied-EPSA FDI performance in CASE1.

F/N Ratio		**1**	**2**	**3**	**4**	**5**	**6**	**7**	**8**	**9**	**10**

Fault detection number		0	63	300	967	1,000	1,000	1,000	1,000	1,000	1,000
Fault isolation number	#1	0	34	165	805	984	999	1,000	1,000	1,000	1,000
	#2	0	8	13	56	15	1	0	0	0	0
	#3	0	14	22	59	14	1	0	0	0	0
	#4	0	29	145	815	985	999	1,000	1,000	1,000	1,000
	#5	0	11	7	7	0	0	0	0	0	0
	#6	0	12	8	10	0	0	0	0	0	0
	#7	0	14	6	9	0	0	0	0	0	0

**Table 5.** Modidfied-EPSA FDI performance in CASE2.

F/N Ratio			**2**	**3**	**4**	**5**	**6**	**7**	**8**	**9**	**10**

Fault detection number		0	0	45	251	746	992	1,000	1,000	1,000	1,000
Fault isolation number	#1	0	0	27	200	675	963	981	992	1,000	1,000
	#2	0	0	38	196	672	960	**1**	992	1,000	1,000
	#3	0	0	7	6	23	24	19	7	0	0
	#4	0	0	5	5	7	1	1	0	0	0
	#5	0	0	3	4	5	2	0	1	0	0
	#6	0	0	5	9	7	2	0	1	0	0
	#7	0	0	2	11	28	25	18	7	0	0
